# Inhibition of 12/15-LOX hyperactivation mitigates cognitive decline in a chronic cerebral hypoperfusion mouse model and in H_2_O_2_-induced HT22 cells: therapeutic effects of brozopine

**DOI:** 10.1080/14756366.2025.2547259

**Published:** 2025-08-21

**Authors:** Xuening Wang, Zhizai Lu, Qiuji Shao, Yi Wang, Zixin Zhang, Zhiyu Wang, Qingran Jia, Jinpeng Zhu, Yiran Song, Lingxu Yuan, Yiming Wang, Shaoyang Xu, Lirou He, Junbiao Chang, Yuan Gao

**Affiliations:** ^a^ Department of Pharmacology, School of Basic Medicine, Zhengzhou University, Zhengzhou, China; ^b^ Institute of Clinical Pharmacology, School of Basic Medicine, Zhengzhou University, Zhengzhou, China; ^c^ Department of Neurosurgery, The Third Affiliated Hospital of Zhengzhou University, Zhengzhou, China; ^d^ Department of Cerebrovascular Disease, Henan Provincial People’s Hospital, Zhengzhou University People’s Hospital, Zhengzhou, China; ^e^ Department of Chemistry and Molecular Engineering, Zhengzhou University, Zhengzhou, China

**Keywords:** BZP, 12/15-LOX, inflammation, CCH, cognitive decline

## Abstract

Brozopine (BZP), a novel inhibitor of 12/15-lipoxygenase (12/15-LOX), has previously demonstrated efficacy in mitigating inflammatory and oxidative stress-related injury in cerebral ischaemia models. This study aimed to evaluate the therapeutic potential and underlying mechanisms of BZP in a mouse model of vascular dementia induced by chronic cerebral hypoperfusion. BZP was administered for 28 days following right unilateral common carotid artery occlusion (rUCCAO) in mice. BZP significantly alleviated cognitive impairment, behavioural deficits, and fine motor function. Mechanistically, BZP inhibited 12/15-LOX, cPLA_2_, p-p38 MAPK/p38 MAPK ratio, tumour necrosis factor-α, interlukin-1β, Aβ_1–42_ deposition, and Tau hyperphosphorylation in the brain and serum of rUCCAO mice. Similar protective effects were observed in both 12/15-LOX-overexpressed and H_2_O_2_-induced HT22 cell models. These findings suggest that BZP exerts its neuroprotective effects by targeting the 12/15-LOX/cPLA_2_/p38 MAPK pathway, offering a promising therapeutic strategy for mitigating the progression of cognitive impairment.

## Introduction

Chronic cerebral hypoperfusion (CCH), resulting from sustained reductions in cerebral blood flow (CBF) due to various causes such as hypertension, diabetes, familial hypercholesterolaemia, and atherosclerosis, leads to progressive brain injury. CCH is widely recognised as a key pathophysiological contributor to cognitive decline, motor dysfunction, and vascular dementia (VD), with its severity closely associated with the severity of dementia. VD – the second most common form of dementia following Alzheimer’s disease (AD) – accounts for 20–25% of all dementia cases and is associated with high mortality and disability rates, imposing a substantial burden on society^1^. Currently, there are no effective therapeutic agents in clinical use to prevent, treat, or halt the progression of VD caused by hypoperfusion. Consequently, identifying new therapeutic targets and developing interventions for CCH-induced VD hold significant clinical and public health value.

12/15-Lipoxygenase (12/15-LOX) is a non-heme iron-containing dioxygenase of the lipoxygenase family, which is highly expressed in the cerebellum, basal ganglia, hippocampus, neurons, and certain glial cells^2^. It is involved in the regulation of cell growth and development, inflammation, and immune responses^3–5^. As a key enzyme in the arachidonic acid (AA) metabolic pathway, 12/15-LOX catalyses the conversion of AA into a series of bioactive inflammatory mediators. Recent studies have implicated 12/15-LOX and its metabolites, including 12-hydroxyeicosatetraenoic acid (12-HETE) and 15-HETE, in the pathogenesis of inflammatory diseases, cardiovascular and cerebrovascular diseases^6^, neurological disorders, and neurodegenerative diseases of the central nervous system^7^. During cerebral ischaemia, elevated AA levels in the brain tissue are associated with increased 12/15-LOX activity and accumulation of 12-HETE and 15-HETE in ischaemic regions^8^. This leads to the activation of leukocytes within blood vessels and the subsequent release of pro-inflammatory mediators that exacerbate tissue injury. Furthermore, elevated levels of 12/15-LOX, 12-HETE, and 15-HETE have been identified in the frontal and temporal lobes and the cerebrospinal fluid of patients with AD^9^. This suggests the involvement of 12/15-LOX and its metabolites in the pathogenesis of AD, leading to learning and cognitive dysfunction. The activation of 12/15-LOX is closely associated with reduced levels of the antioxidant glutathione – an early biomarker of Parkinson’s disease – and its inhibition has been shown to mitigate the neurotoxic effects of nitric oxide^10^. Furthermore, 12/15-LOX and 12-HETE have been shown to directly activate p38 mitogen-activated protein kinase (p38 MAPK) and cytosolic phospholipase A_2_ (cPLA_2_), promoting the phosphorylation of p38 MAPK and contributing to neuroinflammation^11^^,^^12^. Although the role of 12/15-LOX in inflammatory diseases is well established, its specific contribution to the development of CCH-induced VD remains largely unexplored.

Currently, several natural and synthetic 12/15-LOX inhibitors – such as nordihydroguaiaretic acid, baicalein, ML351, and PD146176 – are under investigation. However, the majority of these inhibitors have not been approved for clinical use due to their toxicity and adverse effects, including severe gastrointestinal and hepatorenal toxicity, limiting their clinical application^13^. Brozopine (BZP), a novel class I drug for ischaemic stroke developed by our research team in China, has demonstrated efficacy in molecular docking, *in vivo*, and *in vitro* studies by protecting against transient focal cerebral ischaemia–reperfusion injury in rats and myocardial ischaemia–reperfusion injury in mice via 12/15-LOX inhibition^14^^,^^15^. BZP has successfully completed preclinical, phase I, and phase II clinical trials and is currently undergoing phase III evaluation, demonstrating encouraging results. However, the therapeutic effect of BZP on CCH-induced VD remains unexplored. The right unilateral common carotid artery occlusion (rUCCAO) mouse model reliably induces mild chronic reductions in CBF, white matter (WM) lesions, delayed memory impairment, and neuroinflammation, making it a valuable model for studying the mechanisms underlying the pathogenesis of CCH-induced VD^16^^,^^17^. In this study, we employed the rUCCAO mouse model and H_2_O_2_-induced injury in HT22 cells to evaluate the neuroprotective potential of BZP in mitigating cognitive decline and neuroinflammation in CCH-associated VD.

## Materials and methods

### Reagents

BZP (purity: 99.4%) was synthesised at the College of Chemistry and Molecular Engineering, Zhengzhou University. Baicalein (purity: ≥98%; LOT: 122452) and AACOCF3 (purity: ≥98%; LOT: 438371) were purchased from MedChemExpress Bio-Technology Co., Ltd. (Shanghai, China). Adezmapimod (SB203580; purity: ≥99%; LOT: K08394) was purchased from KKL Med Inc. (Ashland, VA). The enzyme-linked immunosorbent assay (ELISA) kits for ALOX15 were purchased from Wuhan Colourful Gene Biotech Co., Ltd. (Wuhan, China). The 12-HETE ELISA kit was purchased from Shanghai Yuanju Biotechnology Centre (Shanghai, China). The 15-HETE ELISA kit was purchased from Wuhan Fine Biotech Co., Ltd. (Wuhan, China). The p-Tau217 ELISA kit was purchased from Shanghai Enzyme-linked Biotechnology Co., Ltd. (Shanghai, China). The Aβ_1–42_ ELISA kit was purchased from Elabscience Biotechnology Co., Ltd. (Wuhan, China). The primary antibodies included anti-12/15-LOX (Santa Cruz, Santa Cruz, CA, sc-133085), anti-p38 MAPK (SAB, San Diego, CA, 11252), anti-p-p38 MAPK (Proteintech, Rosemont, IL, 28796), anti-tumour necrosis factor (TNF)-α (Proteintech, Rosemont, IL, 17590-1-AP), anti-interleukin (IL)-1β (Proteintech, Rosemont, IL, 26048-1-AP), anti-cPLA_2_ (Proteintech, Rosemont, IL, 68133-1-Ig), anti-total Tau (Abcam, Cambridge, UK, ab254256), anti-Ser396 Tau (Abcam, Cambridge, UK, ab109390), anti-Aβ_1–42_ (Invitrogen, Carlsbad, CA, 44-344), α-tubulin (Abclonal, Woburn, MA, AC012), and glyceraldehyde-3-phosphate dehydrogenase (GAPDH; Goodhere, Hangzhou, China, AB-P-R001). The fluorescence secondary antibodies included Alexa Fluor™ 488 (Thermo Fisher, Waltham, MA, A-11001) and Alexa Fluor™ 594 (Thermo Fisher, Waltham, MA, A-11012). Foetal bovine serum (FBS; LOT: S829012029) was purchased from Invigentech (Irvine, CA). High-glucose Dulbecco’s modified Eagle medium (DMEM; LOT: 10013150) was purchased from Corning (Corning, NY). Tris-buffered saline + Tween (TBST) was obtained from Beijing Leagene Biotech Co., Ltd. (Beijing, China). DMEM (LOT: 240003001) and phosphate-buffered salne (PBS; LOT: 240002003) were obtained from Beijing Solarbio Technology Co., Ltd. (Beijing, China). Trypsin (LOT: DEGA2402011) was purchased from Shanghai Zhong Qiao Xin Zhou Biotechnology Co., Ltd. (Shanghai, China).

### Establishment of the rUCCAO model^18^

Male C57BL/6 mice (age: 6–8 weeks; weight: 19–21 g) were purchased from Liaoning Changsheng Biotechnology Co., Ltd. (Shenyang, China) (certification number: SCXK2020-0002). The mice were housed in a specific pathogen-free animal facility under controlled environmental conditions at 22 ± 2 °C and 50% humidity, under a 12-h light/dark cycle with *ad libitum* access to food and water. They were placed in ventilated cages with 4–5 mice per cage. Prior to the experiment, the mice underwent an acclimatisation period of at least seven days. All experimental procedures were approved by the Animal Care and Use Committee of Zhengzhou University, and were performed following the ARRIVE guidelines for *in vivo* animal experiments.

Following the procedures outlined in a previous study, C57BL/6 mice were anaesthetised using isoflurane (4% for induction and 2% for maintenance). A midline cervical incision was made, and the right common carotid artery was carefully isolated from the vagus nerve. The artery was then double-ligated both proximally and distally to the heart using 6-0 surgical sutures. Subsequently, the skin was sutured and disinfected with iodine. In the sham group, the right common carotid artery was exposed and isolated without ligation. For all mice, the incisions were closed, and they were allowed to recover in a controlled, comfortable environment. After the final behavioural assessment, the mice were anaesthetised with isoflurane inhalation, and 0.5–0.8 mL of blood was collected from the tail vein. Following blood collection, the mice were euthanised, and finally decapitated for brain extraction.

### Morris water maze (MWM) test^19^

The MWM test was conducted to evaluate spatial learning and memory in mice. The maze consisted of a circular water tank measuring 120 cm in diameter and 50 cm in height. A circular platform measuring 10 cm in diameter and 35 cm in height was positioned in the centre of one quadrant and submerged 1.0 cm below the water surface. Over a five-day navigation period, mice underwent daily training to locate the hidden platform by being placed in the water facing the pool wall. The average latency to locate the platform was recorded as the daily learning score. Each training session was limited to 1 min. If a mouse failed to find the platform within this time, it was gently guided to the platform and allowed to remain there for 20 s. On day 6, a probe test was performed with the platform removed. Mice were placed in the water in the quadrant opposite to the location of the original platform, and their movement trajectories, and the number of platform crossings within 1 min were automatically recorded using the ZS-001 Morris-analysis software (Beijing, China) to assess spatial memory.

On the 28th day after rUCCAO surgery, the MWM test was conducted by three blinded observers to evaluate the cognitive function of each ischaemic mouse. Only successful VD mice identified through this process were included in the study. The screening criteria (SC) for VD mice were defined as follows: the average escape latency of each mouse in the rUCCAO group was recorded as Time1, whereas that in the sham group was recorded as Time2. The SC was calculated using the formula: SC = (Time1 − Time2)/Time1 × 100%. An SC score greater than 20% indicated cognitive impairment.

### Experimental design

Male mice were randomly divided into six groups (*n* = 10–11 per group) to investigate therapeutic effect of BZP: sham, rUCCAO, baicalein (30 mg/kg), and BZP (concentration: 7.5, 15, and 30 mg/kg) groups. The dosage of BZP was determined by converting results from the dose–response relationship curve established in our previous study on the therapeutic effects of BZP in tMCAO rats (data unpublished). Baicalein, a classic 12/15-LOX inhibitor, has demonstrated therapeutic potential in ameliorating the severity of various diseases. Therefore, it served as the positive control in the present study, with its dosage selected based on the doses reported by Cui et al.^20^ The sham and rUCCAO groups received daily intravenous injections of an equivalent volume of normal saline, whereas the other groups were administered their corresponding drugs in the same manner for 30 days.

### Cell culture and establishment of the H_2_O_2_-induced HT22 cell oxidative stress model

HT22 cells, an immortalised mouse hippocampal neuron cell line, were obtained from the Shanghai Institute of Cell Biology, Chinese Academy of Sciences, Shanghai, China. The cells were cultured in high-glucose DMEM supplemented with 10% FBS and maintained at 37 °C in a humidified incubator with 5% CO_2_ and 95% air.

HT22 cells were treated with different doses of H_2_O_2_ (300–900 μM) to induce oxidative stress, whereas control cells were treated with PBS (see Supplementary results).

### Cell viability assay

The HT22 cells were seeded in 96-well plates at a density of 6 × 10^3^ cells/well. To investigate the effect of BZP on H_2_O_2_-induced HT22 cells, the cells were pre-treated without and with varying concentrations of BZP for 24 h, followed by exposure to 300 μM H_2_O_2_ for 20 h. Subsequently, the original medium was replaced with a freshly prepared medium containing the CCK8 solution, and the cells were incubated at 37 °C for 1 h after washing with PBS. The absorbance (*A* value) was measured at 450 nm using a microplate reader (ELX800, Bio-Tek, Norcross, GA), with the aim of elucidating the dose–response relationship of BZP. Baicalein served as a positive control, with its concentration determined based on the dose reported by Li et al.^21^.

### Haematoxylin and eosin (H&E) staining

After anaesthetisation, the mice brains were removed, perfused transcardially with saline, and fixed in 4% paraformaldehyde overnight at 4 °C. The brain tissues were then progressively dehydrated and embedded in paraffin. Coronal sections were prepared and immersed in 1% cresyl violet solution at 50 °C for 1 h, followed by dehydration using a graded ethanol series. The sections were subsequently cleared with xylene. Finally, 5-µm-thick tissue sections were stained using an H&E staining kit (Servicebio, Wuhan, China) and a Nissl staining kit (Servicebio, Wuhan, China), following the manufacturers’ instructions. Pathomorphological changes in the hippocampal, cortical, and striatal tissue sections were observed under a microscope (Olympus, Tokyo, Japan).

### Behavioural testing

#### Neurological deficit assessment^22^

Mice were placed in the test chamber for 1 h prior to testing to allow acclimatisation to the environment. Neurological deficits were assessed using the Zea-Longa scoring method as follows: 0: normal; 1: left forelimb weakness, indicating a mild neurological deficit; 2: circling to the left when walking, indicating a moderate neurological deficit; 3: falling to the left when walking, indicating severe neurological deficit; and 4: loss of consciousness. Higher scores correspond to more severe neurological deficits. Two blinded observers performed the neurobehavioural tests.

#### Motor balance and coordination assessment

Motor balance and coordination were evaluated using the beam balance test^23^. The balance beam, measuring 2.5 cm in width, 80 cm in length, and positioned 100 cm above the ground, was set up with one end placed inside a black square box. Mice were positioned at the opposite end of the beam. The scoring criteria were as follows: 0: mice walked symmetrically and maintained balance on the beam; 1: mice primarily relied on the unaffected limbs to traverse the beam; 2: mice predominantly used the unaffected limbs to walk on the beam; 3: mice were incapable of walking on the beam; and 4: mice fell off the beam immediately upon placement.

The forelimb grip strength of mice was assessed using a grip strength metre (Beijing, China)^24^. Each mouse was lifted by the tail and encouraged to grasp a rigid grid connected to a digital force gauge. The tail was then gently pulled backward, and the tension recorded by the digital force gauge was defined as the grip strength prior to the mouse releasing the grid. Five consecutive tests were conducted for each mouse, and the mean maximum limb muscle strength value (in g) was calculated.

The ability of the mice to maintain walking speeds on a rotating rod was assessed using a rotarod apparatus (Beijing, China)^25^. Mice were placed in the lanes of the rotarod with the initial rod speed set at 0 rpm/min. The device was set to acceleration mode, and the speed was gradually increased from 0 to 40 rpm over a period of 5 min. The time at which the mice fell off the rotating rod was recorded. The test was repeated three times with at least 15 min between trials, and the data were reported as the median of three trials.

### Immunofluorescence staining and immunohistochemistry (IHC) analysis

For immunofluorescence staining, the frozen brain tissue was cut into serial coronal sections (40 μm) and washed with PBS. Next, they were incubated with primary antibodies anti-12/15-LOX (1:200; Santa Cruz, Santa Cruz, CA, sc-133085) and anti-Aβ_1–42_ (1:1000; Invitrogen, Carlsbad, CA, 44-344) at 4 °C overnight. Finally, the sections were washed with PBS and incubated with the corresponding fluorescent-labelled secondary antibody for 50 min at 37 °C, followed by washing with PBS. 4′,6-Diamidino-2-phenylindole, a fluorescent stain that binds strongly to the DNA, was used to detect cell nuclei in the sections. The changes in the fluorescence intensity of 12/15-LOX and Aβ_1–42_ in the hippocampus, cortex, and striatum in each group were observed under a fluorescence microscope (Invitrogen, Carlsbad, CA) and analysed and quantified using ImageJ software (Bethesda, MD).

The brain tissue sections were sliced, dewaxed, rehydrated, and subjected to antigen retrieval, followed by blocking with H_2_O_2_. The sections were then incubated overnight at 4 °C with the following primary antibodies: anti-Aβ_1–42_ (1:400; Servicebio, Wuhan, China, GB111197-100), anti-p38 MAPK (1:500; Servicebio, Wuhan, China, GB154685-100), and anti-cPLA_2_ (1:500; Servicebio, Wuhan, China, GB11551-100). Following incubation with the primary antibodies, the sections were further incubated with the appropriate secondary antibody. Subsequently, the frozen sections were dehydrated, counterstained with haematoxylin, cleared in xylene, and mounted with neutral gum. Finally, the sections were observed and imaged under a microscope.

### ELISA

Blood was collected and serum was isolated via centrifugation at 1500 × *g* at 4 °C for 10 min. The levels of Aβ_1–42_ and p-Tau217 were detected according to the manufacturer’s instructions of the respective kits (Elabscience Biotechnology Co., Ltd., Wuhan, China; Shanghai Enzyme-linked Biotechnology Co., Ltd., Shanghai, China).

The mouse brain extracts were collected and mixed with normal saline at a volume ratio of 1:9 (tissue weight:saline volume). The mixture was homogenised in an ice-water bath and then centrifuged at 1000 × *g* for 20 min at 4 °C. The levels of 12/15-LOX, 12-HETE, 15-HETE, and Aβ_1–42_ were detected according to the manufacturer’s instructions of the respective kits (Wuhan Colourful Gene Biotech Co., Ltd., Wuhan, China; Shanghai Yuanju Biotechnology Centre, Shanghai, China; Wuhan Fine Biotech Co., Ltd., Wuhan, China; Shanghai Enzyme-linked Biotechnology Co., Ltd., Shanghai, China; Elabscience Biotechnology Co., Ltd., Wuhan, China).

### RNA extraction and quantitative real-time polymerase chain reaction (RT-qPCR) analysis

For qPCR, the total RNA was extracted from the mouse hippocampus by using the SteadyPure Quick RNA Extraction Kit (Accurate Biotechnology Co. Ltd., Guangzhou, China). The RNA concentration in each group was measured using Thermo Fisher Nanodrop One (Waltham, MA) and recorded. The total RNA was reverse-transcribed into cDNA using the HiScript III RT SuperMix for qPCR kit (Vazyme, Nanjing, China, R323-01), and the reverse-transcribed cDNA was immediately used for qPCR. The relative mRNA expression was assayed using the SYBR qPCR Master Mix kit (Vazyme, Nanjing, China, Q711-02) on a QuantStudio™ Five Flex Real-Time PCR System (Singapore) and calculated using the 2^−ΔΔ^Ct method. The transcription level of GAPDH was used as an internal control for the values representing mRNA expression. The sequences of the PCR primers used in these experiments were as follows:

Mouse-ALOX15: forward 5′-TCTACCCCACCGCCGATTTT-3′
Reverse 5′-AGCCACCAAGTGTCCCCTCA-3′
Mouse-p38 MAPK: forward 5′-GGGGCATCGTGTGGCAGTTA-3′
Reverse 5′-CAGTGACCTTGCGGGTGTGA-3′
Mouse-TNF-α: forward 5′-GACCCTCACACTCACAAACCA-3′
Reverse 5′-TTGTCCCTTGAAGAGAACCTG-3′
Mouse-GAPDH: forward 5′-AGAGTGGGAGTTGCTGTTG-3′
Reverse 5′-GCCTTCCGTGTTCCTACC-3′

### Western blotting

The total protein was extracted from isolated hippocampal tissue or HT22 cells. The protein concentration was quantified using the Pierce BCA Protein Assay Kit (Solarbio, Beijing, China, PC0020). The protein samples were separated on 10% or 12.5% SDS PAGE gels (Epizyme, Cambridge, MA, PG112; PG113) and electrophoretically transferred to polyvinylidene fluoride membranes. The membranes were blocked with 5% non-fat milk in TBST for 1 h at room temperature (25 °C) and then incubated with the primary antibodies – including anti-12/15-LOX (1:300, Santa Cruz, Santa Cruz, CA, sc-133085), anti-p38 MAPK (1:1000, SAB, 11252), anti-p-p38 MAPK (1:1000, Proteintech, Rosemont, IL, 28796), anti-TNF-α (1:1000, Proteintech, Rosemont, IL, 17590-1-AP), anti-IL-1β (1:1000, Proteintech, Rosemont, IL, 26048-1-AP), anti-cPLA_2_ (1:2000, Proteintech, Rosemont, IL, 68133-1-Ig), anti-total Tau (1:3000, Abcam, Cambridge, UK, ab254256), anti-Ser396 Tau (1:3000, Abcam, Cambridge, UK, ab109390), α-tubulin (1:1000, Abclonal, Woburn, MA, AC012), and GAPDH (1:1000, Goodhere, Hangzhou, China, AB-P-R001) – overnight at 4 °C. After washing three times for 5 min with TBST, the membranes were incubated with the appropriate secondary antibody for 2 h at room temperature and finally subjected to ECL chemiluminescence treatment and exposure. ImageJ software (Bethesda, MD) was used for observation and greyscale analyses.

### Lentiviral transfection of HT22 cells

HT22 cells were cultured in high-glucose DMEM (Corning, Corning, NY, 10013150) containing 10% FBS (Invigentech, Irvine, CA, S829012029) and 1% penicillin/streptomycin (Legene, CA0075) in 5% CO_2_ at 37 °C. HT22 cells were initially cultured in 12-well plates, followed by transfection with the 12/15-LOX-overexpressed lentivirus or negative control lentivirus (Genechem Co., Ltd., Shanghai, China). The transfected cells were selected with puromycin (2 μg/mL; Santa Cruz, Santa Cruz, CA) 72 h after transfection. Transfection efficiency was observed under a fluorescence microscope (Invitrogen, Carlsbad, CA) and evaluated using Western blot analysis.

### Statistical analysis

One-way analysis of variance was used to compare the means ± standard deviation among the groups, along with Dunnett’s *post hoc* test, calculated with GraphPad Prism 8.0 software (San Diego, CA). A *p* value of <0.05 was considered statistically significant.

## Results

### BZP improves learning and memory, neurological function, and motor coordination in rUCCAO mice

**Figure 1.**
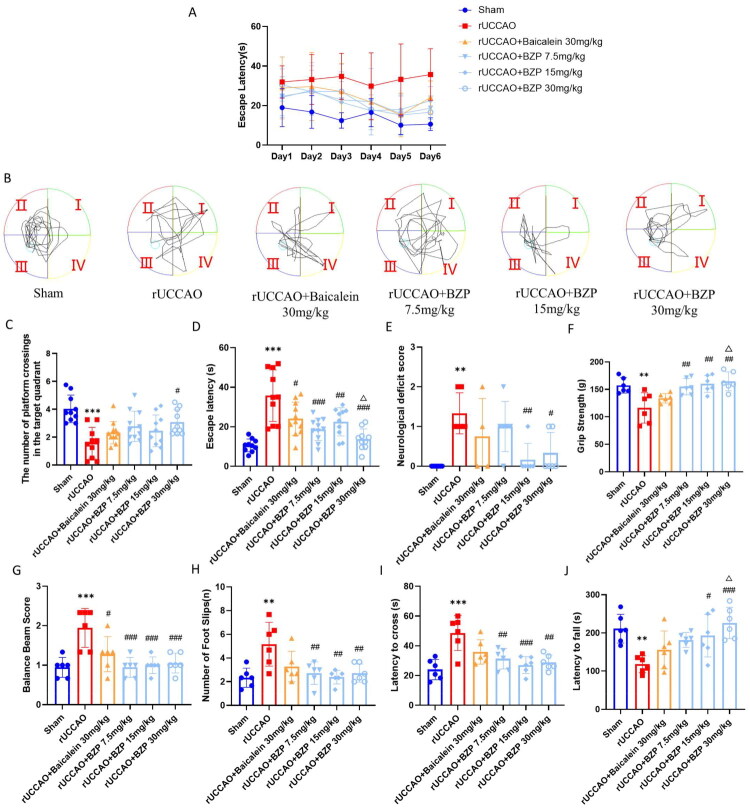
Effect of BZP on the learning and memory performance, neurological function, and fine motor coordination in rUCCAO mice. (A) Escape latency from day 1 to day 6. (B) Representative traces in the MWM test. (C, D) Changes in the number of platform crossings in the target quadrant, and the average time spent in the target quadrant on day 6. (E) Zea-Longa scores. (F) Forelimb grip strength test results. (G) Balance beam scores. (H) Number of foot slips. (I) Latency time to cross the balance beam. (J) Latency time to fall from the rotating rod. Data are expressed as means ± standard deviation. One-way analysis was used to statistical significance (**p < 0.01 , ***p < 0.001 vs. sham group; ^#^*p* < 0.05 vs. rUCCAO group, ^##^*p* < 0.01 , ^###^*p* < 0.001 vs. rUCCAO group; ^△^*p* < 0.05 vs. rUCCAO + baicalein 30-mg/kg group).

To evaluate the neurobehavioural impact of BZP, we established the rUCCAO model and screened for successful induction using the MWM test (see Supplementary results for details). One month post-surgery, we assessed cognitive, neurological, and fine motor functions using the MWM test, Zea-Longa score, balance beam test, and rotarod test. As shown in Figure 1, compared with the sham group, the rUCCAO group exhibited a significantly prolonged average escape latency on day 6 (*p* < 0.01), increased number of neurological deficits (*p* < 0.01), higher balance beam scores and number of foot slips (both *p* < 0.001), increased latency to cross the balance beam (*p* < 0.001), shorter stay time on the rotarod (*p* < 0.01), and diminished forelimb grip strength *p* < 0.01). Treatment with BZP (7.5, 15, and 30 mg/kg) (*F*_(5,56)_ = 13.21; *p* < 0.001, *p* < 0.01, and *p* < 0.001, respectively) or baicalein (30 mg/kg) (*p* < 0.05) significantly reduced the escape latency. Additionally, the rUCCAO + BZP 30-mg/kg group demonstrated an increased number of platform crossings in the target quadrant on the sixth day (*F*_(5,56)_ = 6.58, *p* < 0.05). The rUCCAO + BZP 15- and 30-mg/kg groups showed a significant reduction in the neurological deficit score (*F*_(5,29)_ = 5.85; *p* < 0.01 and *p* < 0.05, respectively). Furthermore, all BZP doses and baicalein improved the grip strength (*F*_(5,30)_ = 7.05, *p* < 0.01, *p* < 0.01, *p* < 0.001, and *p* > 0.05, respectively) and latency to fall from the rotarod (*F*_(5,30)_ = 5.83; *p* > 0.05, *p* < 0.05, *p* < 0.001, and *p* > 0.05, respectively) while reducing balance beam scores (*F*_(5,30)_ = 8.07; *p* < 0.001, *p* < 0.001, *p* < 0.001, and *p* *<* 0.05, respectively), number of foot slips (*F*_(5,30)_ = 5.40; *p* < 0.01, *p* < 0.01, *p* < 0.01, and *p* > 0.05, respectively), and beam-crossing latency (*F*_(5,30)_ = 7.81; *p* < 0.01, *p* < 0.001, *p* < 0.01, and *p* > 0.05, respectively). Interestingly, the rUCCAO + BZP 30-mg/kg group outperformed the rUCCAO + baicalein 30-mg/kg group in reducing escape latency, enhancing grip strength, and prolonging rotarod duration (all *p* < 0.05).

These results indicate that BZP significantly improves learning, memory performance, neurological function, and fine motor coordination in rUCCAO mice, with superior therapeutic efficacy compared with baicalein.

### BZP alleviates histopathological damage in the brain *in vivo* and *in vitro*

HE staining revealed that the hippocampal, cortical, and striatal neurons in the sham group had intact nuclei and organised morphology, whereas the rUCCAO group exhibited nuclear pyknosis, cellular swelling, and increased necrosis. Treatment with BZP (7.5, 15, and 30 mg/kg) and baicalein (30 mg/kg) significantly reduced neuronal necrosis in the hippocampus (*F*_(10,36)_ = 1.21; *p* < 0.001, *p* < 0.001, *p* < 0.001, and *p* *<* 0.01, respectively), cortex (*F*_(10,36)_ = 1.21; *p* > 0.05, *p* < 0.01, *p* < 0.01, and *p* *<* 0.05, respectively), and striatum (*F*_(10,36)_ = 1.21; *p* < 0.01, *p* < 0.01, *p* < 0.01, and *p* > 0.05, respectively). Furthermore, treatment with 15 and 30 mg/kg of BZP showed greater efficacy than the 30 mg/kg of baicalein (both *p* < 0.05) (Figure 2(A,B)).

Figure 2.Effects of BZP on morphological damage in the hippocampus, cortex, and striatum of rUCCAO mice and oxidative stress damage in H_2_O_2_-induced HT22 cells. (A) Representative images of HE staining in the hippocampus, cortex, and striatum. (B) Quantitative analysis of the percentage of deformed and necrotic neurons in the hippocampus, cortex, and striatum. (C) Representative images of Nissl staining in the hippocampal CA1, CA3, and DG regions (scale bars: 275 μm (×20) and 125 μm (×40); *n* = 3/group for HE and Nissl staining, respectively). (D) Bright-field images revealing the loss of HT22 cell viability and alternations in cell morphology following induction with H_2_O_2_, which are remarkably ameliorated by administering BZP (scale bar: 125 μm). (E) Dose–response curve of H_2_O_2_-induced HT22 cell viability: 300 μM of H_2_O_2_ and low-glucose DMEM are added to treat HT22 cells for 20 h, with BZP pre-treatment (1–50 μM) for 24 h. Data are expressed as means ± standard deviation. One-way analysis of variance was used to determine statistical significance (**p < 0.01, ***p < 0.001 vs. sham group/control group; ^#^*p* < 0.05, ^##^*p* < 0.01, ^###^*p* < 0.001 vs. rUCCAO group/H_2_O_2_ group; ^Δ^*p* < 0.05 vs. rUCCAO + baicalein 30-mg/kg group).

**Figure:**
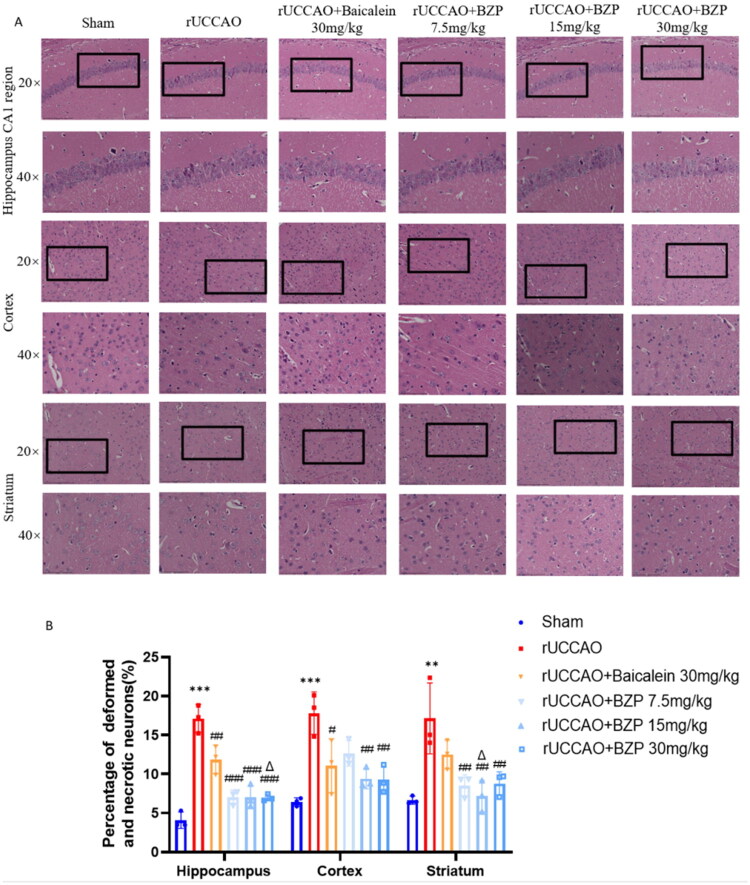

**Figure:**
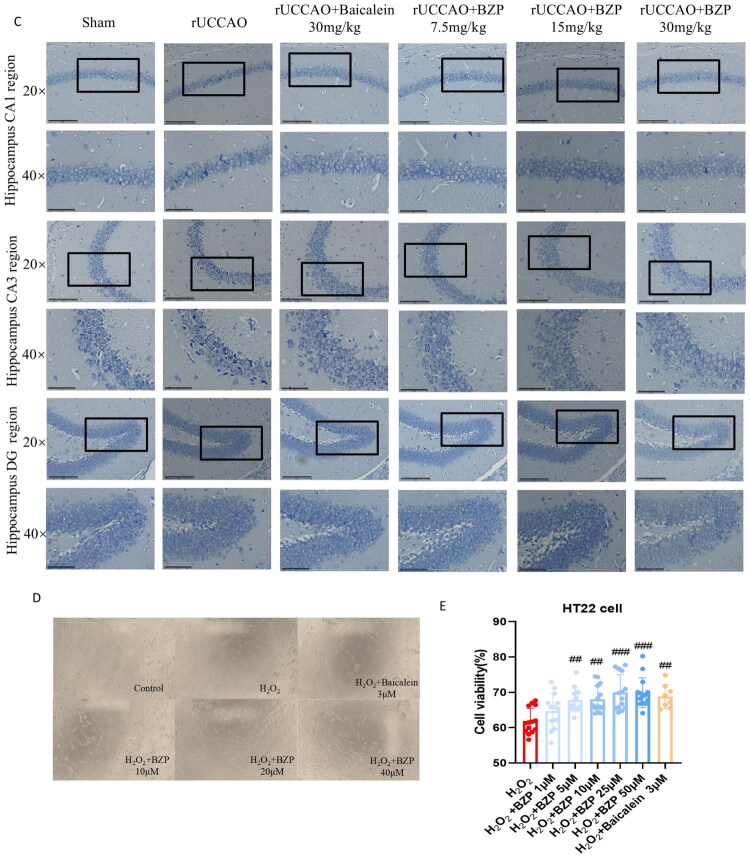

Nissl staining revealed disrupted neuronal arrangement and nuclear condensation in the CA1, CA3, and DG regions of rUCCAO mice. By contrast, baicalein- and BZP-treated mice treatment groups retained more pyramidal neurons with clearer layering and structural integrity in the hippocampal CA1, CA3, and DG regions of rUCCAO mice (Figure 2(C)).

After establishing a stable H_2_O_2_-induced oxidative stress model using HT22 cells and determining the safe concentration range of BZP (data provided in Supplementary results), we constructed a dose–response curve for BZP and calculated its IC_50_ (15.99 µM) against H_2_O_2_-induced HT22 cells. In the control group, HT22 cells exhibited intact morphology with a uniform and densely packed arrangement. By contrast, H_2_O_2_-induced HT22 cells showed reduced cell viability, morphological atrophy, synaptic retraction, and disorganised cell arrangement. Pre-treatment with BZP or baicalein improved the cell morphology and increased the cell viability (*F*_(6,85)_ = 7.19; *p* < 0.01, *p* < 0.01, *p* < 0.001, *p* *<* 0.001, and *p* < 0.01, respectively). Based on these findings, we selected BZP concentrations of 10, 20, and 40 μM for further exploring its protective mechanisms (Figure 2(D,E)).

### Transcriptomic sequencing analysis results

**Figure 3.**
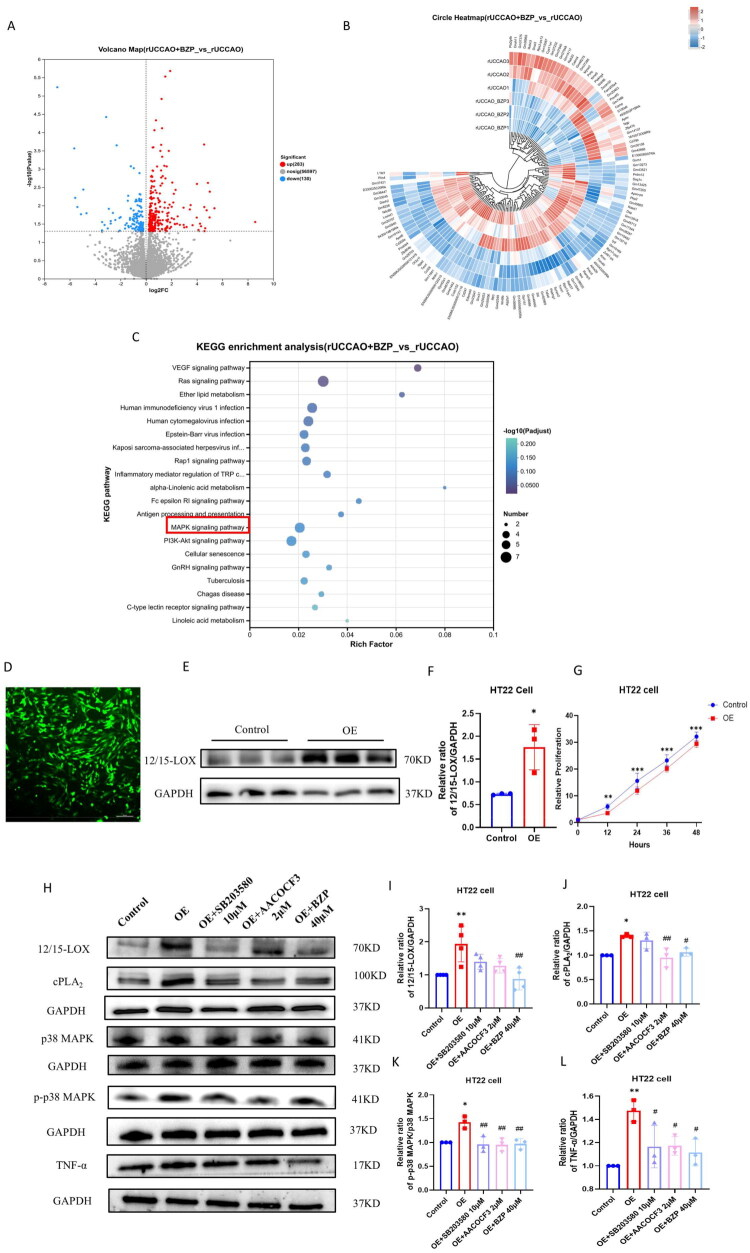
Effects of BZP on the expression of differentially expressed genes (DEGs) in rUCCAO mice. (A) Volcano plot visualising DEGs induced by BZP. The grey dots indicate genes with no significant difference, the red dots indicate upregulated DEGs, and blue dots indicate down-regulated DEGs. (B) Heatmap of DEGs. (C) Significantly enriched KEGG pathway. (D) Transfection of lenti-12/15-LOX into HT22 cells. Fluoresce microscope was used to observe the transfection efficacy of 12/15-LOX-OE-GFP in HT22 cells (×200 magnification). (E, F) Western blot analysis to detect the expression of 12/15-LOX in control and overexpression (OE) groups. (G) Effect of 12/15-LOX OE on the relative proliferation of HT22 cells. (H–L) Protein expression levels of 12/15-LOX, cPLA_2_, p38 MAPK, p-p38 MAPK, and TNF-α in OE, OE + SB203580, OE + AACOCF3, and OE + BZP groups using Western blot analysis. Data are presented as means ± standard deviation (*p < 0.05, **p < 0.01, ***p < 0.001 vs. control group, ^#^*p* < 0.05, ^##^*p* < 0.01 vs. OE group).

Transcriptome analysis of the rUCCAO + BZP (30 mg/kg) group versus the rUCCAO group revealed 413 differentially expressed genes (DEGs), with 283 upregulated and 130 downregulated genes (Figure 3(A,B)). Kyoto Encyclopaedia of Genes and Genomes (KEGG) pathway enrichment identified 20 significantly enriched KEGG pathways, including Herpes simplex virus 1 infection, circadian rhythm regulation, spliceosome, and mitogen-activated protein kinase (MAPK) signalling, in the rUCCAO + BZP 30-mg/kg group compared with those in the rUCCAO group (Figure 3(C)).

To further validate the involvement of the MAPK signalling pathway, we examined the protein expression levels of 12/15-LOX, cPLA_2_, p38MAPK, p-p38MAPK, and TNF-α in 12/15-LOX-overexpressing HT22 cells using Western blot analysis. As illustrated in Figure 3(D–G), the expression level of 12/15-LOX was significantly higher in the overexpression (OE) group than in the control group (*p* < 0.05). However, the relative proliferation rate of cells in the OE group was significantly lower at 12, 24, 36, and 48 h (*p* < 0.01, *p* < 0.001, *p* < 0.001, and *p* < 0.001, respectively).

The expression levels of 12/15-LOX (*p* < 0.01), cPLA_2_ (*p* < 0.05), and TNF-α (*p* < 0.01) were significantly higher in the OE group than in the control group, along with an increased p-p38 MAPK/p38 MAPK ratio (*p* < 0.05).

Furthermore, we observed that BZP treatment significantly decreased the protein expression levels of 12/15-LOX (*F*_(4,15)_ = 6.84, *p* < 0.01), cPLA_2_ (*F*_(4,10)_ = 7.70, *p* < 0.05), p-p38 MAPK/p38 MAPK (*F*_(4,10)_ = 8.38, *p* < 0.01), and TNF-α (*F*_(4,10)_ = 7.52, *p* < 0.05) in OE cells. Similar reductions were observed after treatment with SB203580 (a p38 MAPK inhibitor; *F*_(4,15)_ = 6.84; *p* *>* 0.05; *F*_(4,10)_ = 7.70, *p* *>* 0.05; *F*_(4,10)_ = 8.38, *p* < 0.01; and *F*_(4,10)_ = 7.52, *p* < 0.05, respectively) and AACOCF3 (a cPLA_2_ inhibitor; *F*_(4,15)_ = 6.84, *p* *>* 0.05; *F*_(4,10)_ = 7.70, *p* *<* 0.01; *F*_(4,10)_ = 8.38, *p* < 0.01; and *F*_(4,10)_ = 7.52, *p* < 0.05, respectively).

These results align with sequencing data, which demonstrated that the activation and expression of 12/15-LOX are closely linked to the MAPK signalling pathway, indicating that the MAPK signalling pathway is involved in mitigating the inflammatory response in rUCCAO mice. Specifically, 12/15-LOX, cPLA_2_, and p38 MAPK form a positive feedback loop that amplifies inflammation. Therefore, the 12/15-LOX inhibitor BZP appears to exert its therapeutic effects on cognitive function in rUCCAO mice by inhibiting the 12/15-LOX/cPLA_2_/p38 MAPK positive feedback loop signalling pathway.

### BZP decreases the expression levels of 12/15-LOX, 12-HETE, 15-HETE, TNF-α, and IL-1β *in vivo* and *in vitro*

As shown in Figure 4, ELISA and RT-qPCR analyses consistently demonstrated that the rUCCAO group exhibited increased hippocampal 12/15-LOX expression (both *p* < 0.05) and its metabolites 12-HETE and 15-HETE (both *p* < 0.001) compared with the sham group. Treatment with BZP (7.5, 15, and 30 mg/kg) and baicalein (30 mg/kg) significantly reduced the mRNA expression levels of 12/15-LOX (*F*_(4,20)_ = 3.51; *p* *>* 0.05, *p* < 0.05, *p* < 0.05, and *p* *>* 0.05, respectively), 12-HETE (*F*_(5,30)_ = 9.67; *p* < 0.001, *p* < 0.001, *p* < 0.001, and *p* < 0.001, respectively), and 15-HETE in the hippocampus (*F*_(5,30)_ = 17.39; *p* < 0.001, *p* < 0.001, *p* < 0.001, and *p* < 0.001, respectively).

Figure 4.Reduced levels of 12/15-LOX, 12-HETE, 15-HETE, TNF-α, and IL-1β following treatment with BZP in the hippocampus of rUCCAO mice and H_2_O_2_-induced HT22 cells. (A–C) ELISA results of the levels of 12/15-LOX, 12-HETE, and 15-HETE in the hippocampus of rUCCAO mice. (D, E) Analysis of the mRNA levels of 12/15-LOX and TNF-α via RT-qPCR. (F–I) Analysis of the protein expression of 12/15-LOX, TNF-α, and IL-1β in the hippocampus of rUCCAO mice via Western blot analysis. (J–M) Analysis of the protein expression of 12/15-LOX, TNF-α, and IL-1β in H_2_O_2_-induced HT22 cells via Western blot analysis. Data are expressed as means ± standard deviation (*p < 0.05, **p < 0.01, ***p < 0.001 vs. sham or control group; ^#^*p* < 0.05, ^##^*p* < 0.01, ^###^*p* < 0.001 vs. rUCCAO or H_2_O_2_ group).

**Figure:**
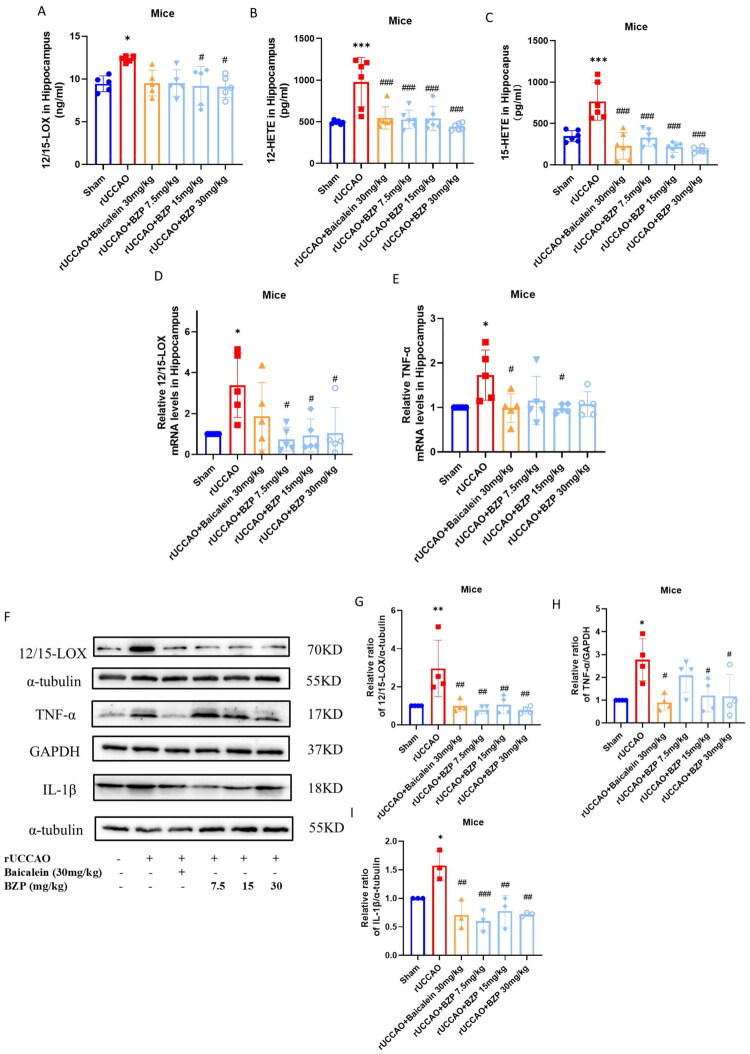

**Figure:**
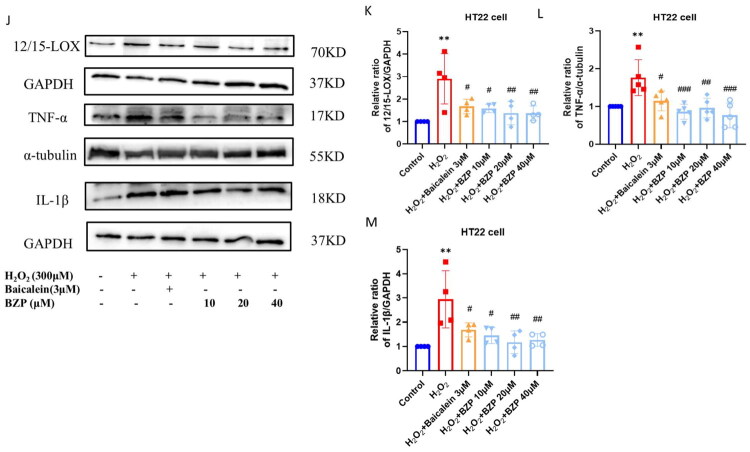

RT-qPCR and Western blot analyses revealed that the mRNA and protein expression levels of TNF-α (both *p* < 0.05) and IL-1β (both *p* < 0.05) were significantly increased in the hippocampus of rUCCAO mice. In contrast, treatment with BZP (15 and 30 mg/kg) and baicalein (30 mg/kg) markedly reduced the mRNA expressions of TNF-α (*F*_(5,24)_ = 3.10; *p* < 0.05, *p* < 0.05, and *p* < 0.05, respectively) and IL-1β (*F*_(5,12)_ = 9.42; *p* < 0.001, *p* < 0.01, *p* < 0.01, and *p* < 0.01, respectively).

Similarly, the protein expressions of 12/15-LOX, TNF-α, and IL-1β (*p* < 0.01, *p* < 0.01, and *p* < 0.01, respectively) were significantly upregulated in the H_2_O_2_ group. In contrast, the H_2_O_2_ + BZP (10, 20, and 40 μM) groups and the H_2_O_2_ + baicalein (3 μM) group demonstrated downregulated protein expression of 12/15-LOX (*F*_(5,18)_ = 5.83, *p* < 0.05, *p* < 0.01, *p* < 0.01, and *p* < 0.05, respectively), TNF-α (*F*_(5,24)_ = 7.50; *p* < 0.001, *p* < 0.01, *p* < 0.001, and *p* < 0.05, respectively), and IL-1β (*F*_(5,18)_ = 6.36; *p* < 0.05, *p* < 0.01, *p* < 0.01, and *p* < 0.05, respectively).

### Effect of BZP on cPLA_2_ and p38 MAPK expressions *in vivo* and *in vitro*

**Figure 5.**
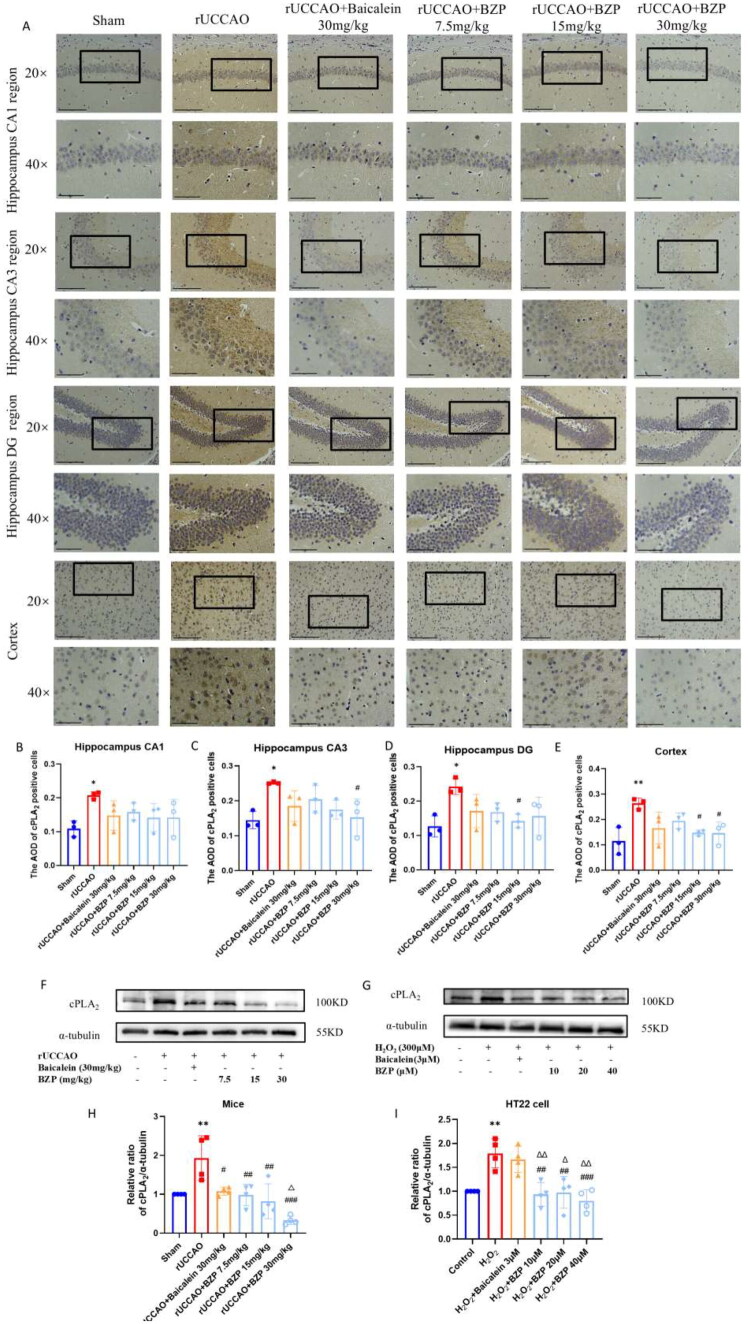
BZP treatment reduces cPLA_2_ levels in the hippocampus and cortex of rUCCAO mice and H_2_O_2_-induced HT22 cells. (A) Representative IHC photomicrographs of cPLA_2_ in the hippocampal CA1, CA3, and DG regions and cortex. (B–E) Quantification of the AOD of cPLA_2_-positive cells in different regions (scale bar: 275 μm (×20) and 125 μm (×40); *n* = 3/group for IHC staining). (F–I) cPLA_2_ protein expression in the hippocampus of rUCCAO mice and H_2_O_2_-induced HT22 cells, as evaluated via Western blot analysis. Data are expressed as means ± standard deviation. One-way analysis of variance was used to determine statistical significance (*p < 0.05, **p < 0.01 vs. sham or control group; ^#^*p* < 0.05, ^##^*p* < 0.01, ^###^*p* < 0.001 vs. rUCCAO or H_2_O_2_ group; ^Δ^*p* < 0.05, ^ΔΔ^*p* < 0.01 vs. rUCCAO + baicalein 30-mg/kg group or H_2_O_2_ + baicalein 3-μM group).

**Figure 6.**
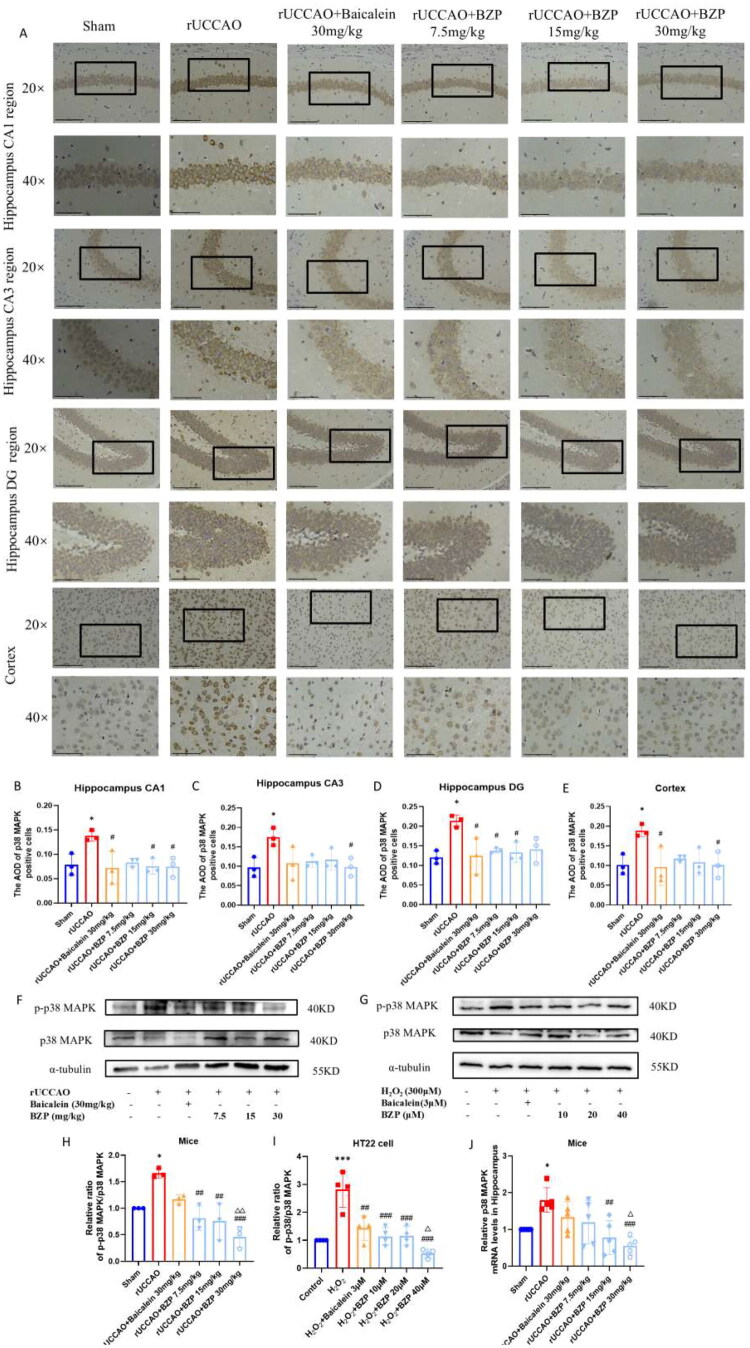
BZP treatment reduces the p-p38 MAPK/p38 MAPK ratio in the hippocampus and cortex of rUCCAO mice and H_2_O_2_-induced HT22 cells. (A) Representative IHC photomicrographs of p38 MAPK in the hippocampal CA1, CA3, and DG regions and cortex. (B–E) Quantification of the AOD of p38-MAPK positive cells in different regions (scale bars: 275 μm (20×) and 125 μm (40×); *n* = 3/group for IHC staining). (F–J) p-p38 MAPK and p38 MAPK protein expression and the p38 MAPK mRNA levels in the hippocampus of rUCCAO mice and H_2_O_2_-induced HT22 cells, evaluated via Western blot and RT-qPCR analyses, respectively. Data are expressed as means ± standard deviation. One-way analysis of variance was used to determine statistical significance (*p < 0.05, ***p < 0.001 vs. sham or control group; ^#^*p* < 0.05, ^##^*p* < 0.01, ^###^*p* < 0.01 vs. rUCCAO or H_2_O_2_ group; ^Δ^*p* < 0.05 vs. rUCCAO + baicalein 30-mg/kg group or H_2_O_2_ + baicalein 3-μM group).

As shown in Figures 5 and 6, IHC, RT-qPCR, and Western blot analyses consistently demonstrated that the expression levels of cPLA_2_ and p38 MAPK, as well as the p-p38 MAPK/p38 MAPK ratio, were significantly increased in the hippocampus and cortex of rUCCAO mice and in the H_2_O_2_ group. Treatment with BZP (7.5, 15, and 30 mg/kg) significantly reduced the expression of cPLA_2_ (*F*_(5,18)_ = 10.38; *p* < 0.01, *p* < 0.01, and *p* < 0.001, respectively) and the p-p38 MAPK/p38 MAPK ratio (*F*_(5,12)_ = 13.42; *p* < 0.01, *p* < 0.01, and *p* < 0.001, respectively), whereas treatment with baicalein (30 mg/kg) reduced only the cPLA_2_ expression (*p* < 0.05). Notably, the reduction in cPLA_2_ levels and the p-p38 MAPK/p38 MAPK ratio were significantly greater following treatment with BZP (30 mg/kg) than with baicalein (*p* < 0.05 and *p* < 0.01, respectively). *In vitro*, BZP (10, 20, and 40 µM) significantly reduced cPLA_2_ expression (*F*_(5,18)_ = 10.84; *p* < 0.01, *p* < 0.01, and *p* < 0.001, respectively) and the p-p38 MAPK/p38 MAPK ratio (*F*_(5,18)_ = 17.54; *p* < 0.001, *p* < 0.001, and *p* < 0.001, respectively), whereas baicalein (3 µM) decreased only the p-p38 MAPK/p38 MAPK ratio (*p* < 0.01). Furthermore, the reductions observed with BZP were more pronounced than those with baicalein.

### Effect of BZP on Aβ_1–42_ levels and tau hyperphosphorylation *in vivo* and *in vitro*

IHC and ELISA results revealed that the relative area of Aβ_1–42_ plaques and its levels in the hippocampus (*p* < 0.05 and *p* < 0.001), cortex (*p* < 0.05), and serum (*p* < 0.01) were significantly higher in rUCCAO mice than in sham mice. Treatment with 30 mg/kg BZP significantly reduced Aβ_1–42_ deposition in the hippocampus and cortex (both *p* < 0.05) and serum (*p* < 0.05), while treatment with 7.5, 15, and 30 mg/kg BZP and 30 mg/kg baicalein significantly decreased Aβ_1–42_ deposition in the hippocampus (*p* < 0.001, *p* < 0.001, *p* < 0.001, and *p* < 0.001, respectively). Interestingly, Aβ_1–42_ levels in serum were also reduced by 30 mg/kg BZP (*p* < 0.05), with a significantly greater reduction than 30 mg/kg baicalein (*p* < 0.001) (Figure 7(A–E)).

Figure 7.BZP treatment reduces Aβ_1–42_ plaque deposition in the hippocampus and cortex, serum Aβ_1–42_ levels, and co-expression of 12/15-LOX and Aβ_1–42_ in the hippocampus, cortex, and striatum of rUCCAO mice. (A) Representative IHC photomicrographs of Aβ_1–42_ in the hippocampus and cortex. (B, C) Quantification of the AOD of Aβ_1–42_-positive cells among the groups. (D, E) Aβ_1–42_ levels in the hippocampus and serum of rUCCAO mice, as evaluated by ELISA. (F) Representative immunofluorescence photomicrographs of 12/15-LOX and Aβ_1–42_ in the (a) hippocampus, (b) cortex, and (c) striatum. (G–L) Relative fluorescence intensity of 12/15-LOX and Aβ_1–42_ in the hippocampus, cortex, and striatum of rUCCAO mice (scale bars: 275 μm (×20) and 125 μm (×40); *n* = 3/group for IHC and immunofluorescence staining). Data are expressed as means ± standard deviation. One-way analysis of variance was used to determine statistical significance (*p < 0.05, **p < 0.01, ***p < 0.001 vs. sham group; ^#^*p* < 0.05, ^##^*p* < 0.01, ^###^*p* < 0.01 vs. rUCCAO group; ^ΔΔΔ^*P* < 0.001 vs. rUCCAO + baicalein 30-mg/kg group).

**Figure:**
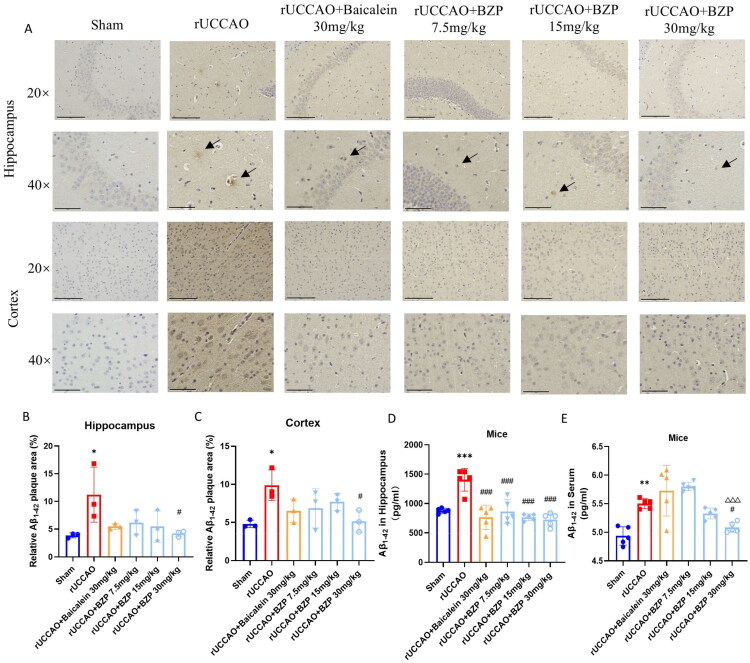

**Figure:**
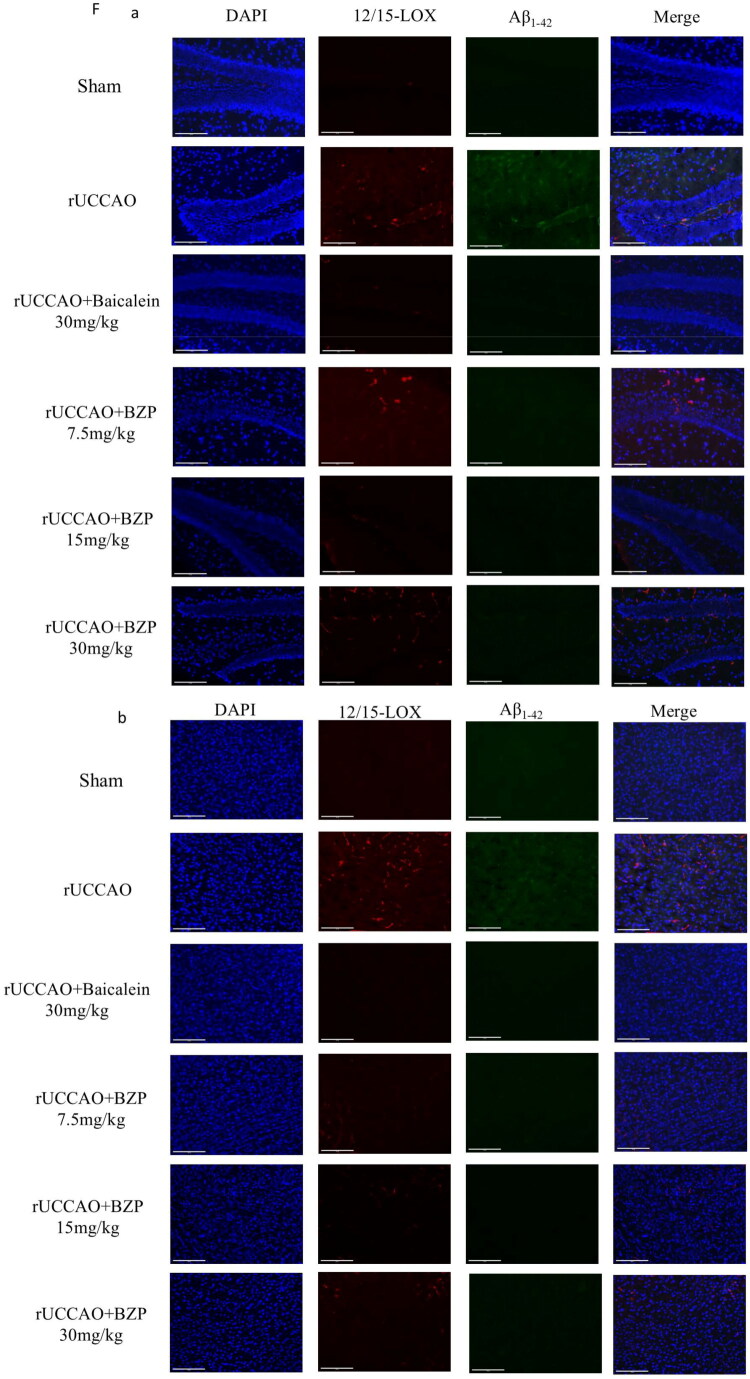

**Figure:**
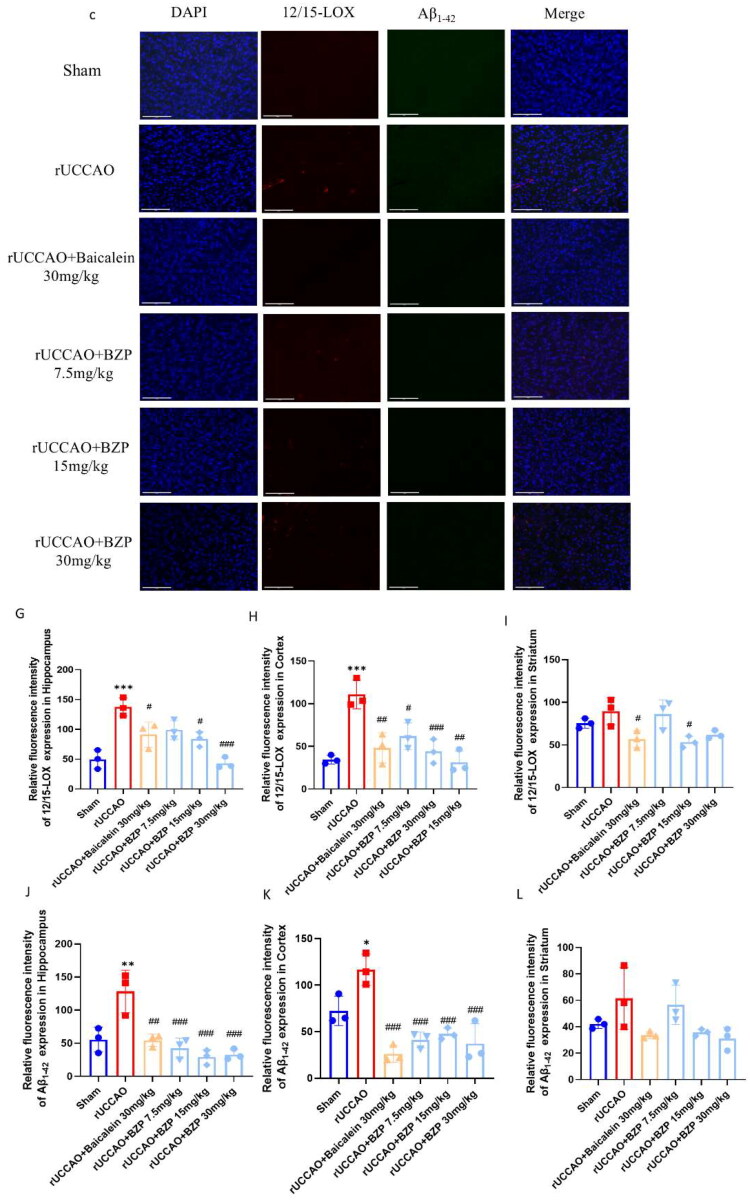

Immunofluorescence staining was performed to evaluate the localised expression of 12/15-LOX and Aβ_1–42_ in the hippocampus, cortex, and striatum of rUCCAO mice. The immunofluorescence intensity of both 12/15-LOX and Aβ_1–42_ in the hippocampus (*p* < 0.001 and *p* < 0.01, respectively) and cortex (*p* < 0.001 and *p* < 0.05, respectively) was significantly greater in rUCCAO mice than in sham mice. However, BZP (7.5, 15, and 30 mg/kg) and baicalein (30 mg/kg) significantly downregulated the immunofluorescence intensity of 12/15-LOX and Aβ_1–42_. Notably, no significant changes were observed in the striatum (Figure 7(F–L)).

**Figure 8.**
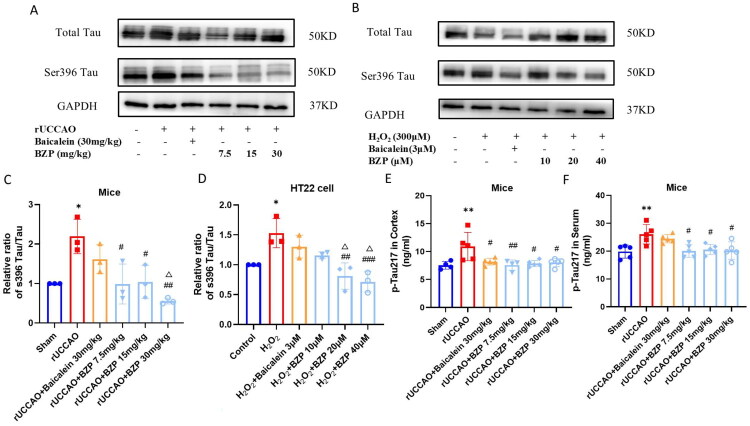
BZP treatment reduces Tau hyperphosphorylation in the hippocampus, cortex, and serum of rUCCAO mice and H_2_O_2_-induced HT22 cells. (A–D) Detection of the Ser396 Tau/total Tau ratio via Western blot analysis. (E, F) Measurement of p-Tau217 levels in the cortex and serum of rUCCAO mice using ELISA. Data are expressed as means ± standard deviation. One-way analysis of variance was used to determine statistical significance (*p < 0.05, **p < 0.01 vs. sham or control group; ^#^*p* < 0.05, ^##^*p* < 0.01 vs. rUCCAO or H_2_O_2_ group; ^Δ^*p* < 0.05 vs. rUCCAO + baicalein 30-mg/kg group or H_2_O_2_ + baicalein 3-μM group).

As shown in Figure 8, Western blot analysis and ELISA demonstrated that the expression of Ser396 Tau/total Tau in the hippocampus (*p* < 0.05) and p-Tau217 in the cortex (*p* < 0.01) were significantly higher in rUCCAO mice than in sham mice. BZP (7.5, 15, and 30 mg/kg) significantly downregulated Ser396 Tau/total Tau expression in the hippocampus (*F*_(5,12)_ = 7.97; *p* < 0.05, *p* < 0.05, and *p* < 0.01, respectively) and p-Tau217 expression in the cortex (*F*_(5,22)_ = 5.01; *p* < 0.01, *p* < 0.05, and *p* < 0.05, respectively) of rUCCAO mice. In HT22 cells, H_2_O_2_ exposure elevated protein expression of Ser396 Tau/total Tau (*p* < 0.05), while BZP (20 and 40 µM) significantly reduced this ratio (*F*_(5,12)_ = 9.54; *p* < 0.01 and *p* < 0.001, respectively). Additionally, Serum p-Tau217 levels were also elevated in rUCCAO mice (*p* < 0.01) but decreased by BZP exposure at all doses (*F*_(5,24)_ = 5.63; all *p* < 0.05).

Collectively, these findings indicate that 12/15-LOX-mediated Aβ_1–42_ accumulation and Tau hyperphosphorylation are alleviated by BZP.

### Significant correlation between 12/15-LOX pathway markers and cognitive impairment indicators in the rUCCAO and rUCCAO + BZP 300 mg/kg groups

**Figure 9.**
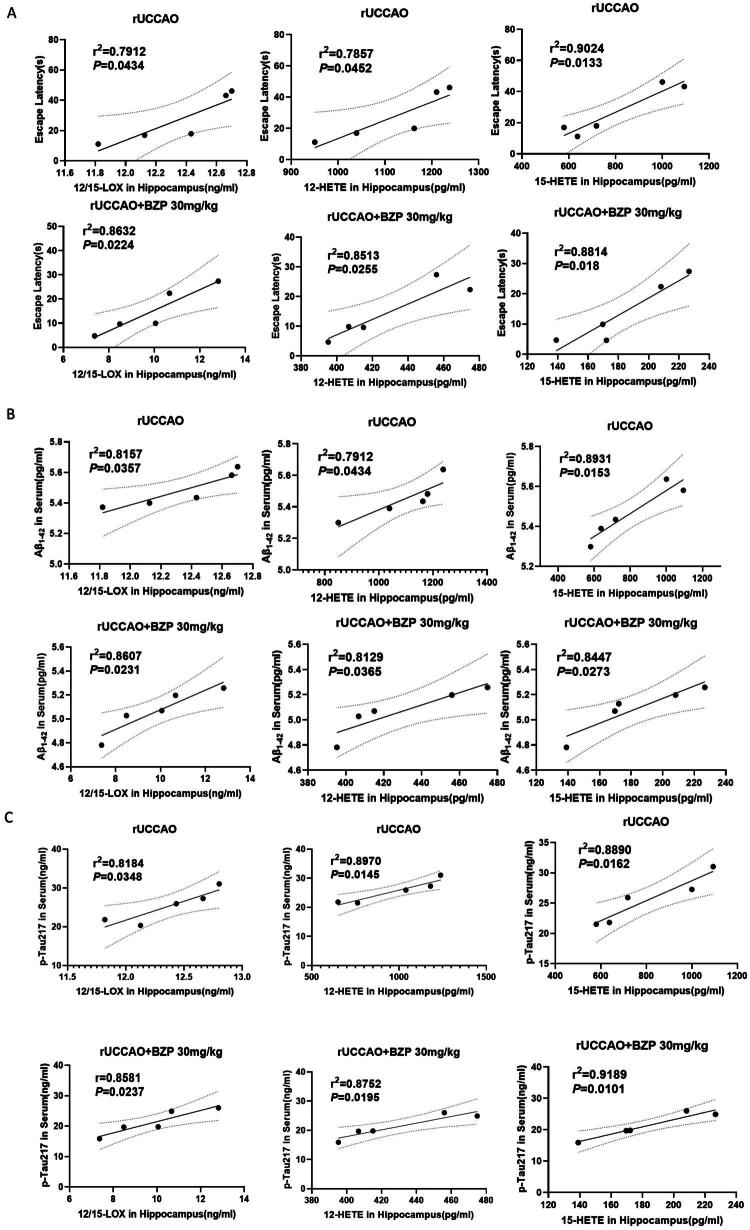
Correlation analysis. (A) Correlation between 12/15-LOX/12-HETE/15-HETE in the hippocampus and the escape latency in rUCCAO mice. (B) Correlation between 12/15-LOX/12-HETE/15-HETE in the hippocampus and Aβ_1–42_ levels in the serum of rUCCAO mice. (C) Correlation between 12/15-LOX/12-HETE/15-HETE in the hippocampus and p-Tau217 levels in the serum of rUCCAO mice. *r*^2^: correlation coefficient. *p* < 0.05 and *p* < 0.01 denote statistical significance.

As shown in Figure 9, correlation analysis revealed that hippocampal levels of 12/15-LOX and its metabolites – 12-HETE and 15-HETE – were positively associated with the escape latency, Aβ_1–42_ levels, and p-Tau217 levels in the serum of both the rUCCAO and rUCCAO + BZP (30 mg/kg) group. These findings suggest that, the higher the levels of 12/15-LOX and its metabolites, the more severe the cognitive impairment.

## Discussion

This study demonstrates that 12/15-LOX plays a pivotal role in the development and progression of CCH-induced VD, and treatment with the 12/15-LOX inhibitor BZP attenuated learning and memory deficits, fine motor dysfunction, neuronal injury, neuroinflammation, and dementia-related pathological markers in both *in vivo* and *in vitro* models. These findings provide the first evidence of the protective effect of BZP against CCH-induced VD, potentially mediated through the suppression of the 12/15-LOX/cPLA_2_/p38 MAPK signalling pathway.

12/15-LOX is a key pro-inflammatory enzyme implicated in several neuroinflammatory disorders^26^. Studies have demonstrated that its metabolites, 12-HETE and 15-HETE, can activate neutrophils and leukocytes and stimulate the release of pro-inflammatory mediators^2^^,^^27^. Conversely, 12/15-LOX inhibition has been shown to alleviate inflammatory responses, such as in allergic airway disease^28^. Neuroinflammation is a critical contributor to cognitive decline^29^, and cytokines such as TNF-α and IL-1β have been shown to enhance the production of amyloid-beta (Aβ) in the brain. The interaction of Aβ with microglia and astrocytes triggers the recruitment of additional inflammatory molecules to the site of inflammation via chemotaxis, thereby amplifying neuroinflammatory responses^30^. Furthermore, excessive production of TNF-α and IL-1β has been strongly associated with impairments in spatial memory^31^. In the present study, rUCCAO mice exhibited significant deficits in cognitive function, neurological performance, and fine motor skills, along with increased hippocampal expression of 12/15-LOX, 12-HETE, and 15-HETE. These changes were positively correlated with the severity of cognitive dysfunction and neurological damage. Furthermore, elevated TNF-α and IL-1β levels further confirmed an activated inflammatory response, aligning with the findings reported by Yosuke et al. BZP treatment substantially ameliorated these pathological features in rUCCAO mice, indicating that BZP mitigates the development of CCH-induced VD by targeting neuroinflammatory mechanisms.

Transcriptomic analysis further supported this mechanism, likely attributed to the enrichment of the MAPK signalling pathway following BZP treatment. Further *in vitro* studies showed that the OE of 12/15-LOX significantly reduced cell proliferation in the OE group and increased the expression of cPLA_2_, p-p38 MAPK/p38 MAPK ratio, and TNF-α. These effects were reduced following treatment with BZP, SB203580 (a p38 MAPK inhibitor), and AACOCF3 (a cPLA_2_ inhibitor), suggesting that the protective effect of BZP is attributed to the disruption of the 12/15-LOX-driven inflammatory signalling cascade.

cPLA_2_ promotes the production of pro-inflammatory factors that trigger the synthesis of eicosanoids, which exacerbate inflammatory reactions^32^. Following cerebral ischaemia, the mRNA expression of cPLA_2_ is significantly upregulated, particularly in the hippocampal CA1–CA3 regions and the dentate gyrus. IL-1β and TNF-α can inhibit both NMDA-dependent and independent long-term potentiation (LTP) in the dentate gyrus of the hippocampal CA1 region in rats via the p38 MAPK pathway, underscoring the central role of p38 MAPK in synaptic dysfunction induced by pro-inflammatory cytokines^33^. The p38 MAPK signalling pathway is closely linked to both 12/15-LOX and cPLA_2_^34^_._ Upon exposure to oxidative or inflammatory stimuli, the p38 MAPK signalling pathway becomes activated, which then enhances cPLA_2_ activity and expression by directly or indirectly targeting the specific amino acid sites on cPLA_2_. This promotes the release of more AA, further amplifying the inflammatory response and indirectly influencing the production of 12/15-LOX. In turn, 12/15-LOX and its metabolites can activate cPLA_2_ and p38 MAPK, which further induce the phosphorylation and the subsequent generation of AA, thus forming a positive feedback loop^35^. Within this network, cPLA_2_ serves as a substrate supplier, bridging the membrane phospholipid metabolism with the 12/15-LOX pathway, whereas p38 MAPK acts as a signalling hub that regulates cPLA_2_ activity and is modulated by feedback from 12/15-LOX metabolites, playing a critical role in cellular signal transduction and pathophysiological processes. Our results confirm this mechanistic interaction. rUCCAO mice showed significant elevations in 12/15-LOX, cPLA_2_, and p-p38 MAPK/p38 MAPK in the hippocampus, cortex, and striatum, all of which were ameliorated by BZP. These findings suggest that BZP disrupts the 12/15-LOX/cPLA2/p38 MAPK feedback loop, thereby reducing neuroinflammation and cell injury.

The p38 MAPK cascade is activated in response to Aβ_1–42_ deposition and tau protein hyperphosphorylation^36^. Once activated, p38 MAPK upregulates β-secretase activity, promoting the conversion of amyloid precursor protein to Aβ, thereby increasing Aβ production. The aggregation of Aβ can induce neuroinflammation and activate the 12/15-LOX/cPLA_2_/p38 MAPK signalling pathway, which further promotes tau protein phosphorylation. This forms a vicious cycle that sustains neurodegeneration. Chen et al.^37^ demonstrated that the 12/15-LOX pathway promotes inflammation and oxidative stress damage by activating p38 MAPK, contributing to diabetic brain injury. They reported that targeting the 12/15-LOX significantly reduces Aβ levels and apoptotic proteins, thereby improving the cognitive function in diabetic brain injury models. The findings of our study are in line with those reported by Chen et al.^37^. In this study, rUCCAO mice exhibited significant Aβ deposition plaques and tau protein hyperphosphorylation in the hippocampal and cortical regions compared with sham mice. Immunofluorescence co-staining further revealed a positive correlation between 12/15-LOX and Aβ_1–42_ expression in rUCCAO mice, along with an increased number of necrotic neurons. Additionally, the serum levels of dementia markers, including Aβ_1–42_ and p-Tau217, were significantly higher in rUCCAO mice than in sham mice, confirming systemic pathological progression. Similar findings were observed in the H_2_O_2_-induced HT22 cells. BZP treatment reversed Aβ_1–42_ deposition, p-Tau217 levels, and Ser396 Tau/total Tau ratio and improved cognitive function, neuronal function, and fine motor coordination in rUCCAO mice. Together, these findings suggest that BZP mitigates VD progression by targeting the 12/15-LOX/cPLA_2_/p38 MAPK pathway, offering a promising therapeutic strategy for VD.

However, several questions remain. The mechanism by which BZP regulates 12/15-LOX expression in neurons and glial cells warrants further investigation, especially in primary cell cultures. Although BZP has progressed to phase III clinical trials and demonstrates promising therapeutic effects, its long-term efficacy and safety still need to be established.

**Figure 10.**
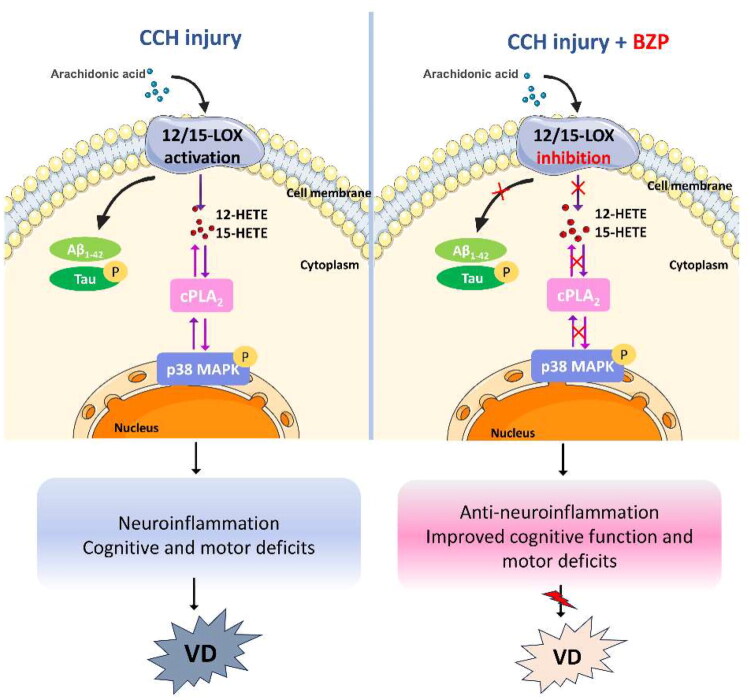
Schematic illustration depicting that 12/15-LOX, cPLA_2_, and p38 MAPK form a positive feedback loop that amplifies inflammation in chronic cerebral hypoperfusion (CCH)-induced vascular dementia (VD). Brozopine (BZP) inhibits 12/15-LOX/cPLA_2_/p38 MAPK signalling in rUCCAO mice, which is largely attributed to the anti-inflammatory properties of BZP achieved via 12/15-LOX inhibition.

In conclusion, this study provides the first evidence that BZP exerts neuroprotective effects in CCH-induced VD by inhibiting the 12/15-LOX/cPLA_2_/p38 MAPK signalling pathway in rUCCAO mice (Figure 10). These anti-inflammatory actions highlight the potential of BZP as a novel therapeutic candidate for VD.

## Supplementary Material

supplement results.doc

## Data Availability

The data that support the findings of this study are available from the corresponding author, Yuan Gao (gaoyuan816@126.com), upon reasonable request.
