# Supplementary material for: Inhibition of 12/15-LOX hyperactivation mitigates cognitive decline in a chronic cerebral hypoperfusion mouse model and in H2O2-induced HT22 cells: therapeutic effects of brozopine
Source: J Enzyme Inhib Med Chem. 2025 Aug 21;40(1):2547259. doi: 10.1080/14756366.2025.2547259 (PMC12372482; doi:10.1080/14756366.2025.2547259)
Supplement: supplement results.doc [file IENZ_A_2547259_SM0762.doc]

**1. CCH successfully induced learning deficit in rUCCAO model.**

On the 26-29th days after the rUCCAO surgery, VD mice were screened using the MWM test. As shown in Supplementary Figure 1, escape latency (time to find the hidden platform) progressively decreased on day 26-30 in all the mice. However, rUCCAO mice had longer escape latencies than the Sham group throughout the training period, demonstrating that chronic cerebral hypoperfusion successfully induced learning deficits in the rUCCAO model. Mice with memory deficit were used in the next step (n = 82). After the first Morris water maze experiment, we rejected unsuccessful rats (n = 25). As a result, 57 mice were used to do experiment.

Supplementary Figure 1 Average escape latency from start point to hidden platform before drug administration in Sham group and rUCCAO group. rUCCAO group were screened by water maze test in fifth week after rUCCAO operation.Data were presented as mean±SD. One-way ANOVA test was used to determine statistical significance. ^*^*P*<0.05, ^***^*P*<0.001*vs*. Control group.

2. Determination of Time and Concentration for H_2_O_2_-Induced Oxidative Stress Injury Model in HT22 Cells and Assessment of Safe Concentration of BZP

As shown in Table 1 and Figure 2, after incubation with various concentrations of H_2_O_2_ for 20-24h, the viability of HT22 cells was determined using a CCK8 assay. The results showed a cell viability of approximately 66.1% (P < 0.001) in 300 μM H_2_O_2_-treated HT22 cells at 20 h, whereas BZP alone (≤200 μM) exhibited a minimal effect on the HT22 cell viability at 24 h compared with the Control group.

Table 1 Effects of H_2_O_2_ treatment with different concentration and time on the survival rate of HT22 cells(mean ± SD, n=3)

| Group | Concentration (µmol/L) |  | 20 h | |  | | 24 h | | |
| --- | --- | --- | --- | --- | --- | --- | --- | --- | --- |
|  |  |  | A450 | Cell  Survival rate(%) | |  | | A450 | Cell  Survival rate(%) |
| Control |  |  | 1.04±0.06 |  |  | | | 1.01±0.04 |  |
| H_2_O_2_ | 300 |  | 0.68±0.02*** | 66.1 |  | | | 0.60±0.02*** | 51.6 |
|  | 400 |  | 0.64±0.02*** | 61.6 |  | | | 0.58±0.03*** | 49.6 |
|  | 500 |  | 0.62±0.05*** | 53.8 |  | | | 0.54±0.03*** | 44.9 |
|  | 600 |  | 0.58±0.06*** | 49.4 |  | | | 0.46±0.03*** | 35.9 |
|  | 700 |  | 0.48±0.04*** | 37.8 |  | | | 0.39±0.02*** | 28.2 |
|  | 800 |  | 0.42±0.02*** | 30.9 |  | | | 0.31±0.02*** | 18.3 |
|  | 900 |  | 0.37±0.02*** | 25.4 |  | | | 0.21±0.01*** | 6.6 |

Note: Effects of H_2_O_2_ treatment with different concentration and time on the survival rate of HT22 cells. Data were presented as mean±SD (n=3). One-way ANOVA test was used to determine statistical significance. ****P*<0.001*vs*. Control group.

Supplementary Figure 2. Determination of safe concentration of BZP and establishment of H_2_O_2_-induced oxidative stress damage model. (A) BZP (1-200 μM) cytotoxicity to HT22 cells was analyzed via CCK-8 assays for a 24 h period.
